# Development of a multiplex real-time PCR for the simultaneous detection of monkeypox virus clades I, II, and goatpox virus

**DOI:** 10.3389/fvets.2024.1483653

**Published:** 2024-11-14

**Authors:** Yongqiang Lin, Zijing Guo, Jinsong Chen, Xianwen Zhang, Long Zhou, Yanmin Li, Zhidong Zhang

**Affiliations:** Key Laboratory of Animal Medicine of Sichuan Province, College of Animal and Veterinary Sciences, Southwest Minzu University, Chengdu, China

**Keywords:** monkeypox virus (MPXV), goatpox virus (GTPV), clade I, clade II, D14L gene, D18L gene, multiplex real-time PCR

## Abstract

**Introduction:**

Monkeypox virus (MPXV) hosts are of multiple species, with a risk of cross-species transmission. This phenomenon poses a threat to unreported affected domestic animals and increases the risk to human public health. Clinical diagnostics continue to face challenges regarding specificity among poxviruses. The need for a rapid and precise assay to differentiate between MPXV clades I and II, as well as goatpox virus (GTPV) is essential for enhancing our capacity for disease prevention, control, and epidemiological investigation.

**Methods:**

To address this need, we have successfully developed a multiplex real-time PCR assay targeting MPXV D14L gene for clade I, MPXV D18L gene for clade II, and GTPV RPO30 gene, which can simultaneously detect MPXV clades I and II as well as GTPV.

**Results:**

The developed assay demonstrated high sensitivity, with limits of detection at 207.83 copies/reaction for MPXV clade I, 252.07 copies/reaction for MPXV clade II, and 208.72 copies/reaction for GTPV. Importantly, there was no cross-reactivity with other non-pox viruses which infect goats. The assay exhibited excellent repeatability, with coefficients of variation (CV%) for intra-assay and inter-assay ranging from 0.17% to 0.89% and 0.58% to 1.09%, respectively.

**Discussion:**

This assay can serve as a vital resource to safeguard against the MPXV epidemic posing a threat to the life safety of goats, to mitigate potential risks to the sheep farming industry, and to prevent the transmission of MPXV to humans through sheep, which could act as a potential transmission vector for infection.

## 1 Introduction

Monkeypox (MPX) is a zoonotic disease caused by MPX virus (MPXV). Since the World Health Organization (WHO) declared MPX outbreak a Public Health Emergency of International Concern in 2022, the situation has been closely monitored. Based on the latest reports from the WHO, from 01 January 2022 to 31 August 2024, there have been 106,310 confirmed cases of MPX across 123 countries and territories, with 234 deaths attributed to the virus (1). Currently, there is a lack of effective treatment options for MPX (2).

MPXV belongs to the family *Poxviridae*, the subfamily of chordate poxviruses, and the Orthopoxvirus genus. MPXV shares ~90% genetic homology with other notable poxviruses, including Variola virus (smallpox), Cowpox virus (CPXV), and Vaccinia virus (VACV) (3–5). MPXV is a double-stranded DNA virus with a genome size of ~197 kilobases (6–8). Currently, it is divided into two clades: clade I (Congo Basin branch) and clade IIa/b (West African branch). Although the genetic sequences of these clades differ by only about 0.5%, they exhibit significant differences in pathogenicity. Given the challenges in diagnosing MPX based solely on clinical signs and postmortem lesions (9, 10). Laboratory tests are essential for definitive diagnosis by detection of MPXV in clinical samples. Several diagnostic methods are available, including virus isolation, pathogenic analysis, serological methods and polymerase chain reaction (PCR) (11, 12). Among these methods, real-time qPCR assays have become the preferred method for the rapid detection and differentiation of MPXV clades I and II, as they are currently the only tests recommended by the WHO for Mpox diagnosis (13, 14).

Unlike the Variola virus, which exclusively affects humans, MPXV has a tendency toward multi-species hosts, Its primary hosts are non-human primates and rodents, but recent reports also include infections in dogs, cats, and pigs which has close contact with human (15–17). This trend suggests that the range species of animals susceptible to MPXV is broad and continues to expand, posing a significant risk of cross-species transmission and a high likelihood of new susceptible animal emerging. Furthermore, this phenomenon also poses a substantial threat to domestic animals not yet reported as infected (18, 19). Therefore, it is very necessary to explore whether MPXV circulating in other domestic animals which have close contact with human, such as cattle, goats, and sheep's (17, 20).

The global goat population has experienced a significant increase over the past decade and ~96% of these animals being meat goats are found primarily in developing countries in Asia and Africa according to Food and Agriculture Organization Statistics in 2017 (21, 22). MPX has reported in many countries in Asia and Africa. In Africa, goats are deeply entrenched in almost every African culture. Goatpox virus (GTPV) primary infect goats and cause serious and contagious skin diseases. This infection is characterized with poxviral lesion that may affect the skin, mucous membranes and internal organs bearing a strong resemblance to the symptoms of MPXV infection. Given close relationship between humans and goats, along with the high genetic similarity and similar symptoms between MPXV and the Goatpox virus (GTPV), raises significant concerns about the risk of spillover infections. Therefore, it is critical not only to differentiate MPXV two clades from each other, but also to distinguish MPXV from GTPV which can infect goats. To address these challenges, specific primers and probes for MPXV clade I and clade II were designed based on MPXV D14L and D18L gene, respectively, and specific primers and probe targeting GTPV RPO30 gene were selected for GTPV from a previous publication (Wang et al.) which was fully validated. After optimizing reaction system, a multiplex real-time PCR assay has been successfully developed. This assay enables simultaneous detection and differentiation of MPXV-I, MPXV-II, and GTPV, which is crucial for the prospective prevention and control of MPX outbreaks and for the differential diagnosis of MPXV and GTPV. The development of such diagnostic tools is particularly important given the complex epidemiology of emerging MPXV, which require rapid differential diagnosis.

## 2 Materials and methods

### 2.1 Virus, primers, and probes

**Figure 1:**
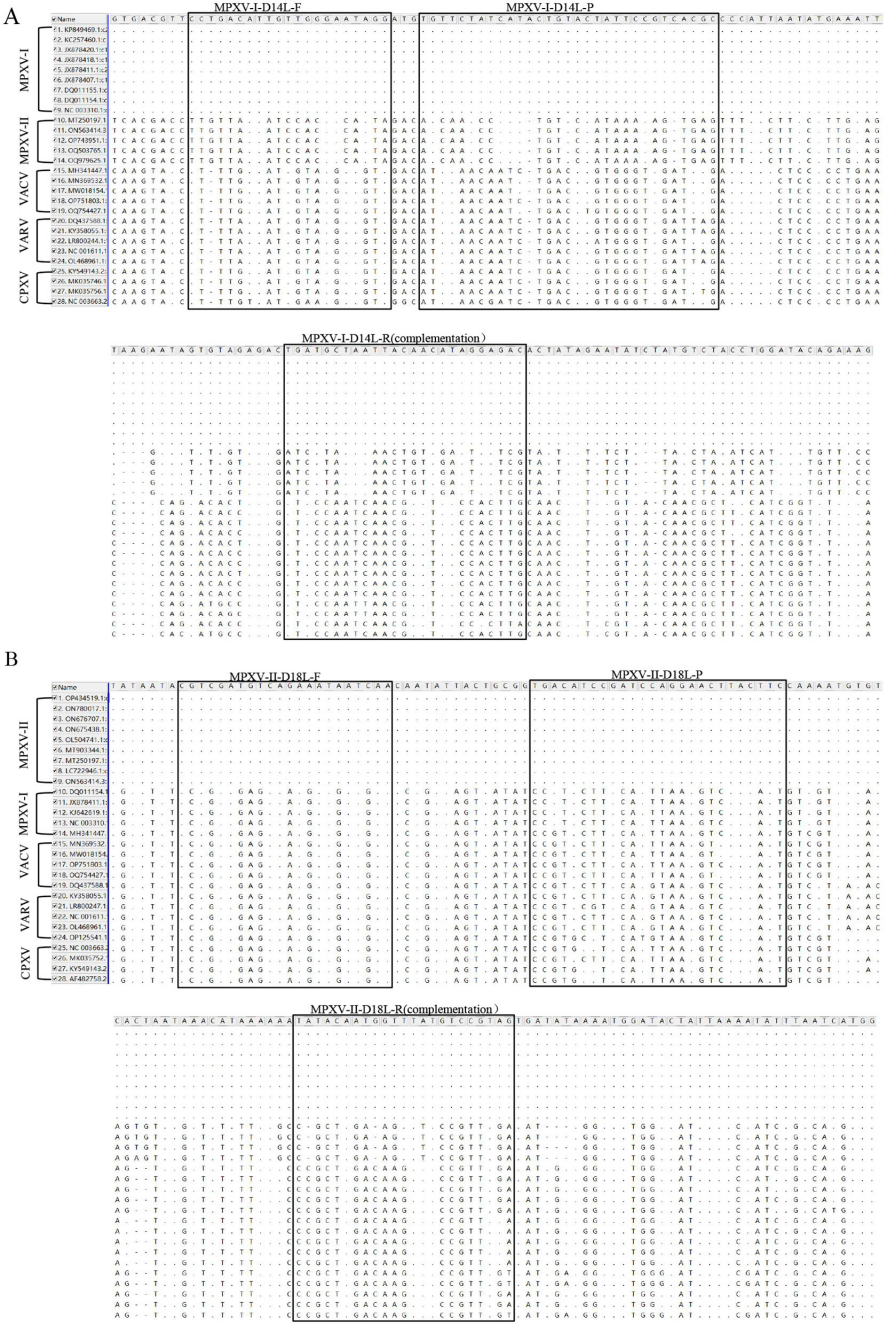
Comparison of target sequences of MPXV and viruses of the same genus. The position of MPXV-I-D14L primer probe was compared with MPXV clade II, VACV, VARV, and CPXV nucleotide sequences **(A)**. The position of MPXV-II-D18L primer probe was compared with MPXV clade I, VACV, VARV, and CPXV nucleotide sequences **(B)**. F, R, and P represent positive and negative primers and TaqMan probes, respectively. “·” represents the same nucleotide, “-” represents A loss of nucleotide, and mutations are noted as “A,” “T,” “C,” or “G”.

The nucleotide sequences for the target fragments of MPXV-I-D14L and MPXV-II-D18L were synthesized by Suzhou Hongxun Biotechnology Company Limited (Suzhou, China). Foot-and-mouth disease virus type A/O (FDMV type A RE-A/WH/09 strain and type O 0/MYA98/BY/2010 strain) vaccine was purchased from Zhongmu Industrial Compane Limited (Beijing, China). GTPV (CVCC AV41 strain) vaccine was obtained from Harbin Pharmaceutical Group Biological Vaccine Company Limited, China. Peste des petits ruminants virus (PPRV) vaccine strain, ORF virus (ORFV), and Mycoplasma pneumoniae (CCPP) were kept in the laboratory. We conducted a comparative analysis of the nucleotide sequences of D14L and D18L genes against other orthopoxviruses available from the GenBank. Specific primers and corresponding probes targeting the highly conserved regions of the D14L and D18L genes were designed using Beacon Designer 8.0 (Table 1). The locations of these primers and probes are shown in Figures 1A, B. Primers and probe for GTPV were selected based on literature reports (23).

**Table 1:** Primers and probes designed in this study.

**Name**	**Sequence (5' to 3')**	**Tm/°C**	**Product/bp**
D14L-F	CCTGACATTGTTGGGAATAGG	54	115
D14L-R	GTCTCCTATGTTGTAATTAGCATCA	53	
D14L-P	Cy5-TGTTCTATCATACTGTACTATTCCGTCACGC-BHQ2	63	
D18L-F	CGTCGATGTCAGAAATAATCAA	50	115
D18L-R	CTACGGACATAAACCATTGTATA	49	
D18L-P	FAM-TGACATCCGATCCAGGAACTTACTTC-BHQ1	61	
Uf-61275	ATGGTAGGATAGTCGCAAATGAT	55	135
Ur-61354	AGATATAAACCCGGCAAGTGAC	57	
P1-61302	HEX-TAAGCGATTTTATAGTTGCAATGCGTAGT–BHQ1	61	

### 2.2 Samples and nucleic acid extraction

Sheep tissues and nasal swabs were collected from sheep farms, slaughterhouses, and individual farmers in Sichuan, China between January 2022 and December 2023 after the first MPX case in humans was reported in China. Tissue samples from golden monkey including heart, lung, kidney, and tumor tissues, were kindly provided by Prof. Hou Rong of Northwestern University, China. The tissue samples were added to 500 μL of phosphate-buffered saline (PBS, pH 7.2) and homogenized by a frozen tissue grinder. The resulting homogenate was subjected to three cycles of freeze-thawing, followed by centrifugation at 12,000 rpm for 5 min. Nasal swabs were processed by adding 500 μL of PBS and vortexing for 2 min. Total nucleic acids from the supernatant (200 μL) and PPRV, A/O FMDV preparations were extracted using TaKaRa MiniBEST Viral RNA/DNA Extraction Kit Ver. 5.0 (TaKaRa, Beijing, China) and GeneJET™ Viral DNA and RNA Purification Kit (Thermo Fisher Scientific, Beijing, China). RNA extracted from PPRV and A/O FMDV preparations were reverse transcribed into complementary DNA (cDNA) using ReverTra Ace qPCR RT Master Mix with gDNA Remover kit (Thermo Fisher Scientific, Beijing, China).

### 2.3 Optimization of reaction conditions

The multiplex real-time PCR reaction systems and procedures were optimized in accordance with the manufacturer's instructions for Taq Pro U+ Multiple Probe qPCR Mix (Novozymes, Nanjing, China). In this experiment, primers volume ranged from 0.2 to 2.0 μL (0.1–1 μM), probes concentration ranged from 0.1 to 0.5 μL (0.05–0.25 μM), and the range of the annealing temperature ranged from 56 to 61°C. The multiplex real-time PCR amplification was conducted using ArchimedX4 system, a Medical fluorescence quantitative PCR instrument from Kunpeng Gene Technology Company Limited (Beijing, China).

### 2.4 Generation of standard curve

MPXV-I-D14L and MPXV-II-D18L plasmids were synthesized by Suzhou Hongxun Biotechnology Company Limited and subsequently cloned into the PUC 57 vector. Additionally, a fragment of GTPV RPO30 gene was amplified and cloned into a pMD 19T vector, resulting in the GTPV-RPO30 plasmid. To determine the copy number of the plasmid, the concentration was converted using the following formula:


$$\begin{array}{l} plasmid\;copies/\mu L=\frac{\left(6.02\;\times \;10^{23}\right)\;\times \;\left(X\left(ng/uL\right)\;\times {\;10}^{-9}\;\right)}{\left(\text{plasmid length }\times \;660\right)} \end{array}$$

The three standard plasmids (MPXV-I-D14L, MPXV-II-D18L, and GTPV-RPO30) were 10-fold serially diluted (ranging from 1.5 × 10^7^ to 1.5 × 10^0^ copies/μL) and equal volumes of each plasmid were mixed in a 1:1:1 ratio. This mixture served as the template for generating the standard curve of the multiplex real-time PCR assay.

### 2.5 Analysis of specificity and sensitivity

For the specificity analysis of the developed multiplex real-time PCR, DNA from ORFV and CCPP as well as cDNA from PPRV and A/O FMDV RNA were used as templates, with ddH_2_O included as a negative control. The clinical manifestations after infection by the aforementioned virus are similar to those of MPXV and GTPV. To evaluate sensitivity, three standard plasmids (MPXV-I-D14L, MPXV-II-D18L, and GTPV-RPO30) were subjected to a 10-fold serial dilution ranging from 1.5 × 10^7^ to 1.5 × 10^−1^ copies/μL. The limit of detection (LOD) for the developed multiplex real-time PCR was determined using these 10-fold serially diluted standard plasmids.

### 2.6 Repeatability analysis

The three standard plasmids (MPXV-I-D14L, MPXV-II-D18L, and GTPV-RPO30) were used at concentrations of 1.5 × 10^4^, 1.5 × 10^3^, and 1.5 × 10^2^ copies/μL to assess the intra-assay and inter-assay variability. Three replicate experiments (three repetitions for each test) were conducted for each dilution and the coefficient of variation (CV%) of the Ct values was calculated.

### 2.7 Evaluation of sample testing

The multiplex real-time PCR assay developed was validated using a variety of sample types, including sheep tissue, nasal swabs and monkey tissue samples as described above. To ensure a comprehensive comparison, the assay was also tested against assays published previously: the multiplex real-time PCR assay for MPXV clades I and II developed by Huo et al. (24), and multiplex real-time PCR assay for goat pox or sheep pox virus by Wang et al. (23). Due to lack of MPXV positive sample, 40 spiked samples with viral standard plasmids were prepared as follows: all of these samples used was detected and confirmed negative for MPXV clades I, II, and GTPV by multiplex real-time PCR assay published previously (24). Five samples were spiked with GTPV plasmids, 16 samples spiked with MPXV clade I and MPXV clade II plasmids, seven samples spiked with MPXV clades I and II plasmids, 10 samples spiked with either MPXV clade I and GTPV plasmids or MPXV clade II and GTPV plasmids, and two samples spiked with MPXV clades I, II and GTPV plasmids. The concentrations of viral standard plasmids blended into tissue samples and nasal swabs were 1.5 × 10^6^, 1.5 × 10^4^, and 1.5 × 10^2^ copies/μL. This process generated a set of positive samples to evaluate the applicability and accuracy of the developed multiplex real-time PCR.

## 3 Results

### 3.1 Determination of optimal reaction conditions

The TaqMan probes for MPXV-I-D14L, MPXV-II-D18L, and GTPV-RPO30 were labeled with Cy5, FAM, and HEX at the 5′ end, and with BHQ2, BHQ1, and BHQ1 at the 3′ end, respectively (Table 1). Primers and probes were synthesized by Sangon Biotech (Shanghai, China). The optimal reaction system was as follows: in a total volume of 20 μL, the reaction mixture included 10 μL of 2 × Taq Pro U+ Multiple Probe qPCR Mix, 0.4 μL of each prime (10 μM), 0.2 μL of each probe (10 μM), 3 μL of nucleic acid template, and 4 μL of enzyme-free water. The amplification conditions were set at 37°C for 2 min, followed by 95°C for 30 s, and then 45 cycles of 95°C for 10 s, and 60°C for 30 s. A CT value of <35 cycles was judged as positive.

### 3.2 Standard curves

**Figure 2:**
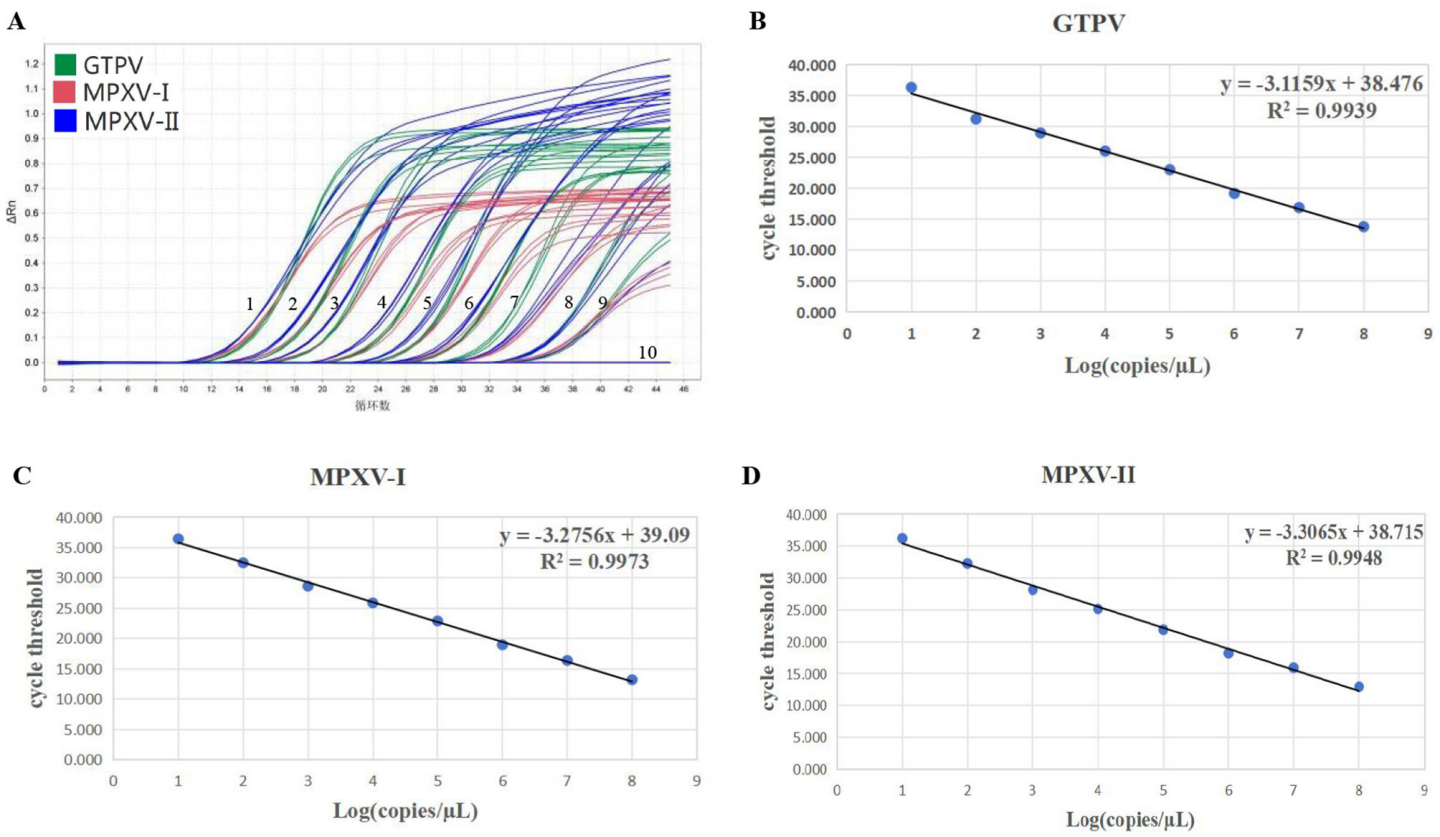
Sensitivity verification and standard curve generation. Amplification curve **(A)** for GTPV, MPXV clade I, and MPXV clade II. Standard curve **(B)** for GTPV, standard curve **(C)** for MPXV clade I, and standard curve **(D)** for MPXV clade II. 1–9: Final concentration of standard plasmid 1.5 × 10^7^-1.5 × 10^−1^ copies/μL, 10: negative control (no enzyme water).

The standard curves for the multiplex real-time PCR assay were generated using three standard plasmids ranging from 1.5 × 10^7^ to 1.5 × 10^0^ copies/μL. The amplification efficiencies, slopes and correlation coefficients (*R*^2^) for MPXV-I, MPXV-II and GTPV were found to be 101.99%, −3.275 and 0.997, 100.67%, −3.306 and 0.994, and 109.42%, −3.115 and 0.993, respectively (Figures 2B–D). The amplification efficiency of the developed multiplex real-time PCR assay fell within the acceptable range of 90–110% and the correlation coefficients complied with 0.99, indicating a strong linear relationship.

### 3.3 Specificity and sensitivity

**Figure 3:**
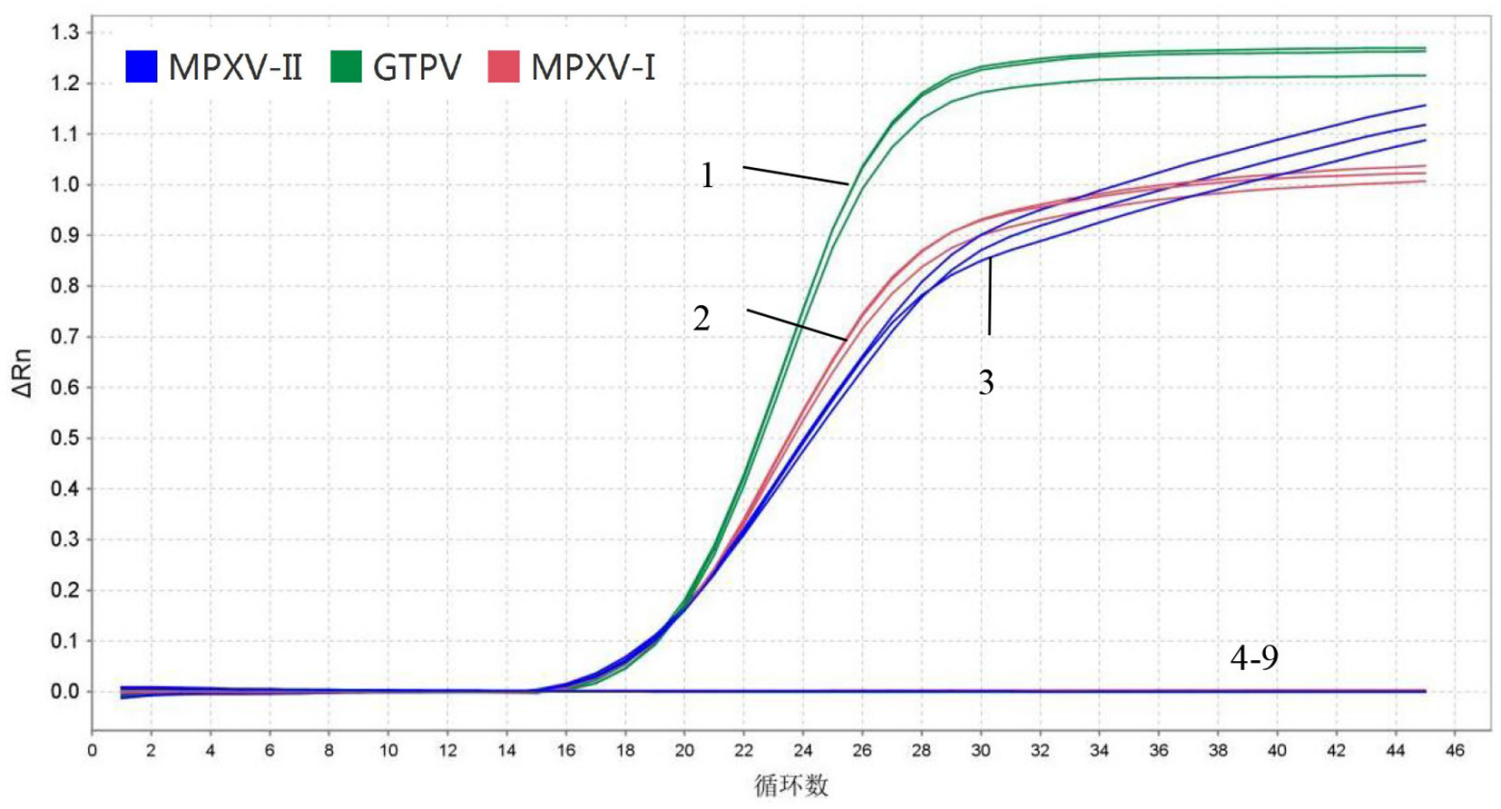
Specificity. 1–3: 1.5 × 10^6^ copies/μL MPXV-I-D14L, MPXV-II-D18L, and GTPV-RPO30 standard plasmids, 4–8: ORFV, A/O type FMDV, PPRV, and CCPP; 9: Negative control (no enzyme water).

In specificity analysis, 1.5 × 10^6^ copies/μL of standard plasmids (MPXV-I-D14L, MPXV-II-D18L, and GTPV-RPO30), DNA from ORPV and CCPP and cDNA from PPRV and FMDV A/O were used. The results showed that MPXV-I-D14L, MPXV-II-D18L, and GTPV-RPO30 exhibited amplification curves while the other viruses exhibited no amplification curves. Importantly the fluorescent signals of MPXV-I, MPXV-II, and GTPV did not interfere with one another, indicating that the developed multiplex real-time PCR assay had good specificity (Figure 3).

For sensitivity testing, standard plasmids (MPXV-I-D14L, MPXV-II-D18L, and GTPV-RPO30) were 10-fold serially diluted, ranging from 1.5 × 10^7^ to 1.5 × 10^−1^ copies/μL. The results showed that the LOD of the developed multiplex real-time PCR assay for detection of MPXV-I, MPXV-II, and GTPV was 1.5 × 10^1^ copies/μL (Figure 2A). Assessing the sensitivity of each primers/probe set alone, the results showed that the LOD for detection of MPXV-I, MPXV-II, and GTPV was 1.5 × 10^0^ copies/μL, 1.5 × 10^1^ copies/μL, and 1.5 × 10^0^ copies/μL, respectively (Supplementary Figure 1). The assay's accuracy was further evaluated with the standard plasmids at a concentration of 300–30 copies/reaction (Table 2). Analysis showed 208.72 copies/reaction (95% confidence interval 180.16–237.27 copies/reaction), 207.83 copies/reaction (95% confidence interval 148.26–267.39 copies/reaction) and 252.07 copies/reaction (95% confidence interval 188.97–315.17 copies/reaction) for GTPV-ORF30, MPXV-I-D14L, and MPXV-II-D18L, respectively.

**Table 2:** The accuracy of LOD by the developed multiplex real-time PCR.

**Plasmid name**	**Copies/ reaction**	**Number of samples**	**Multiplex real-time PCR**	
			**Ct (average)**	**Accuracy rate**
GTPV-ORF30	300	15	32.12	100%
	250	15	33.14	100%
	200	15	33.81	100%
	150	15	34.93	66.7%
	100	15	35.77	23.3%
	30	15	36.86	0%
MPXV-I-D14L	300	15	32.63	100%
	250	15	33.47	100%
	200	15	33.94	100%
	150	15	34.78	60%
	100	15	35.30	16.7%
	30	15	36.20	0%
MPXV-II-D18L	300	15	32.81	100%
	250	15	33.53	100%
	200	15	34.40	86.7%
	150	15	34.85	53.3%
	100	15	35.77	16.7%
	30	15	36.47	0%

### 3.4 Repeatability

To evaluate the repeatability of the developed multiplex real-time PCR assay, three different concentrations of standard plasmids (1.5 × 10^4^, 1.5 × 10^3^, and 1.5 × 10^2^ copies/μL) were tested. The results showed that the coefficients of variation (CV%) for intra- and inter assay variation ranged from 0.17 to 0.89% and 0.58 to 1.09%, respectively (Table 3).

**Table 3:** Intra—and inter-assay repeatability of the developed multiplex real-time PCR.

**Virus target gene name**	**Copy number (copies/μL)**	**Intra-assay**		**Inter-assay**	
		$\bar{\text{X}}$±**SD**	**CV (%)**	$\bar{\text{X}}$±**SD**	**CV (%)**
GTPV-ORF30	1.5 × 10^2^	28.82 ± 0.05	0.169	28.59 ± 0.23	0.802
	1.5 × 10^3^	26.06 ± 0.12	0.460	25.90 ± 0.19	0.617
	1.5 × 10^4^	22.59 ± 0.05	0.239	22.54 ± 0.13	0.662
MPXV-I-D14L	1.5 × 10^2^	28.38 ± 0.15	0.521	28.29 ± 0.17	0.743
	1.5 × 10^3^	25.99 ± 0.23	0.867	25.92 ± 0.28	1.091
	1.5 × 10^4^	22.12 ± 0.13	0.567	22.24 ± 0.19	0.785
MPXV-II-D18L	1.5 × 10^2^	28.24 ± 0.09	0.317	28.40 ± 0.19	0.577
	1.5 × 10^3^	25.71 ± 0.07	0.275	25.74 ± 0.20	0.860
	1.5 × 10^4^	21.93 ± 0.07	0.340	22.08 ± 0.16	0.721

### 3.5 Test results with animal samples

A total of 249 sheep tissue samples, nasal swab and monkey tissue samples, along with 40 spiked samples were tested using the developed multiplex real-time PCR assay and the assays published previously [Huo et al. for MPXV clades I and II (24) and Wang et al. for GTPV (23)]. Real-time PCR for GAPDH gene were used to validate negative results (Supplementary Figure 2). As shown in Table 4, the developed multiplex real-time PCR assay showed that 248 tissue samples and nasal swabs were negative (no amplification curve/Ct > 35), while 40 spiked samples and one sheep nasal swab sample were positive for GTPV (Ct <35). Sanger sequencing results confirmed that the nasal swab sample was tested positive for GTPV. The published assays revealed that 249 tissue samples and nasal swabs and 14 spiked samples were negative (no amplification curve, Ct > 35) and 26 spiked samples were positive (Ct <35). The detection results of MPXV clades I and II in the 14 spiked samples were all negative. The samples were spiked with the standard plasmid at a concentration of 1.5 × 10^2^ copies/μL. Based on the sample testing data and the results from the spiked samples (Table 4), the detection sensitivity of the developed triple qPCR assay significantly surpassed that of the published method. The developed multiplex real-time PCR assay demonstrated a clinical sensitivity and specificity of 100%, while the published assay had a relative sensitivity of 63.42% and a clinical specificity of 100%. In the performance comparison between the developed multiplex real-time PCR and the published real-time PCR detection approaches for detecting MPXV I and II as well as GPTV in clinical and spiked samples, the relative sensitivity of the developed multiplex real-time PCR method amounted to 157.69%, while the relative specificity reached 100% (Table 5).

**Table 4:** Comparison between the developed multiplex real-time PCR and the published assays on clinical samples and spiked samples.

**Sample**	**Spiked with viral genome (copy number)**		**Sample type (number)**	**Assays**				**Agreement**
				**Multiplex real-time PCR**		**Published assays**		
Spiked sample	GTPV	1.5 × 10^6^	Sheep tissues (*n* = 1) and nasal swabs (*n* = 2)	3/3 (100%)		3/3 (100%)		100%
		1.5 × 10^2^	Sheep tissues (*n* = 1) and nasal swabs (*n* = 1)	2/2 (100%)		2/2 (100%)		
	MPXV-I	1.5 × 10^6^	Sheep tissues (*n* = 1) and nasal swabs (*n* = 2)	3/3 (100%)		3/3 (100%)		75%
		1.5 × 10^4^	Golden monkey tissues (*n* = 3)	3/3 (100%)		3/3 (100%)		
		1.5 × 10^2^	Sheep tissues (*n* = 1) and nasal swabs (*n* = 1)	2/2 (100%)		0/2 (0%)		
	MPXV-II	1.5 × 10^6^	Sheep tissues (*n* = 1) and nasal swabs (*n* = 2)	3/3 (100%)		3/3 (100%)		75%
		1.5 × 10^4^	Golden monkey tissues (*n* = 3)	3/3 (100%)		3/3 (100%)		
		1.5 × 10^2^	Sheep tissues (*n* = 1) and nasal swabs (*n* = 1)	2/2 (100%)		0/2 (0%)		
	MPXV-I, MPXV-II	1.5 × 10^6^	Sheep tissues (*n* = 1) and nasal swabs (*n* = 1)	MPXV-I (2)	2/2 (100%)	MPXV-I (2)	2/2 (100%)	71.43%
				MPXV-II (2)		MPXV-II (2)		
		1.5 × 10^4^	Golden monkey tissues (*n* = 3)	MPXV-I (3)	3/3 (100%)	MPXV-I (3)	3/3 (100%)	
				MPXV-II (3)		MPXV-II (3)		
		1.5 × 10^2^	Sheep tissues (*n* = 1) and nasal swabs (*n* = 1)	MPXV-I (2)	2/2 (100%)	MPXV-I (0)	0/2 (0%)	
				MPXV-II (2)		MPXV-II (0)		
	MPXV-I, GTPV	1.5 × 10^6^	Sheep tissues (*n* = 1) and nasal swabs (*n* = 1)	MPXV-I (2)	2/2 (100%)	MPXV-I (2)	2/2 (100%)	40%
				GTPV (2)		GTPV (2)		
		1.5 × 10^2^	Sheep tissues (*n* = 1) and nasal swabs (*n* = 2)	MPXV-I (3)	3/3 (100%)	MPXV-I (0)	0/3 (0%)	
				GTPV (3)		GTPV (3)		
	MPXV-II, GTPV	1.5 × 10^6^	Sheep tissues (*n* = 1) and nasal swabs (*n* = 1)	MPXV-II (2)	2/2 (100%)	MPXV-II (2)	2/2 (100%)	40%
				GTPV (2)		GTPV (2)		
		1.5 × 10^2^	Sheep tissues (*n* = 1) and nasal swabs (*n* = 2)	MPXV-II (3)	3/3 (100%)	MPXV-II (0)	0/3 (0%)	
				GTPV (3)		GTPV (3)		
	MPXV-I, II, GTPV	1.5 × 10^2^	Sheep nasal swabs (*n* = 2)	MPXV-I (2)	2/2 (100%)	MPXV-I (0)	0/2 (0%)	0%
				MPXV-II (2)		MPXV-II (0)		
				GTPV (2)		GTPV (2)		
Field sample	Non		Sheep nasal swab (*n* = 249)	GTPV (1)	1/249 (0.40%)	Non	0/249 (0%)	99.60%
Total			289			289		

**Table 5:** Performance of the developed multiplex real-time PCR in comparison with the real-time PCR assay published for detecting MPXV I and II as well as GPTV on clinical samples and spiked samples.

		**Published methods**			**Performance characteristics (%)**	
		**Positive (MPX and GPTV)**	**Negative (MPX and GPTV)**	**Total (*****n*** = **289)**	**Sensitivity**	**Specificity**
Multiplex real-time PCR	Positive (MPX and GPTV)	26	15	41	157.69%	100%
	Negative (MPX and GPTV)	0	248	248		
	Total (*n* = 289)	26	263	289		

## 4 Discussion

Recent epidemiological studies and data from WHO have indicated that since 2022 MPXV clade II has emerged as the predominant epidemic strain globally, posing a more significant threat than MPXV clade I, which is predominantly endemic in African regions (25, 26). To date, the host-spectrum of MPXV infections and the identity of reservoir hosts remain incompletely understood (27). The increasing number of MPX host species has been reported, with transmission to dogs via humans in both France and Brazil in 2022 and MPXV infection in pigs in Congo (16). This underscores the need to understand the emergence of MPXV spillover risk to humans and animals.

Given the close interaction between humans and goats, along with the high genetic homology and similar clinical symptoms between MPXV and GTPV, there is a urgent need for a rapid and accurate assay for simultaneous detection and differentiation of MPXV clades I, II, and GTPV. There are several routine diagnostic methods for identifying MPXV and GTPV, primarily including pathogenic, serological and molecular assays. Pathogenic testing is one of the most traditional and reliable methods; however, it is not suitable for rapid diagnosis due to its extended test duration, high operational risks and stringent laboratory requirements. Serological methods primarily detect IgM and IgG antibodies, which are relatively easy to perform and applicable in a variety of contexts. However, the presence of immune cross-reactivity among orthopoxviral genera and poxviruses complicates the differentiation between specific poxviruses as well as between antibodies produced through viral infection and those generated by active immunization. Molecular diagnostic techniques, such as real-time PCR methods, loop-mediated isothermal amplification (LAMP) and recombinase-mediated isothermal amplification (RAA), offer convenience and speed, and have been developed for detection of MPXV or GTPV (13, 14). In real-time quantitative PCR for MPXV, the target genes primarily include B6R, B7R, F3L, N3R, J7R, O2L, G2RG, G2R, _WA, C3L, A4L, A27L, A29L, B2R, E9L, and J2R genes, while the isothermal amplification technique mainly targets MPXV A27L, C3L/D14L, J2R, D18L, and F3L genes (13, 14). It was found that the isothermal amplification technology based on the MPXV D14L and D18L genes proves to be superior to that based on other genes. In this study, we analyzed the D14L and D18L genes and designed new primers and probes specific for MPXV clades I and II. Its LOD for detecting MPXV-I, MPXV-II and GTPV reached 10.4, 10.35, and 12.6 copies/μL, respectively. Kong et al. (28) established a real-time PCR method for detection of MPXV, reporting an LOD of 36.5 copies/μL. Additionally, Huo et al. (24) developed a real-time PCR assay for detection and differentiation of MPXV clades IIa, IIb, and I clades as well as the B.1 lineage with LODs of 183 and 119 copies/μL for MPXV clades I/II, respectively. Therefore, the sensitivity of the developed multiplex real-time PCR assay targeting D14L for MPXV I and D18L genes for MPXV II proves to be superior to current qPCR methods based on other MPXV genes.

The sensitivity of the singleplex setting is higher than that of the multiplex setting. It is important to analyze the influence of the coexistence of high and low concentrations of the pathogen on the sensitivity before the assay is used for diagnostic purposes. Evaluation with animal samples and spiked samples showed that the developed multiplex real-time PCR assay is specific and sensitive, and can simultaneously detect and differentiate MPXV-I, MPXV-II, and GTPV, indicating its potential as a novel diagnostic assay for diagnosis of MPXV-I, MPXV-II, and GTPV in the field. This method, as an entirely new technical support, can achieve accurate differential diagnosis of GTPV and MPXV in clinical applications. Importantly, it offers powerful technical support for investigating the host source of MPXV and potential intermediate hosts, and for studying the cross-species transmission mechanism of MPXV, contributing to the potential risk prediction and epidemiological research of MPXV in the future. To facilitate the use of this assay in the field, a larger number of animal samples are required to validate the assays before such an assay can be considered for routine diagnostic use.

## 5 Conclusion

In this study, we established a multiplex real-time PCR assay for faster, sensitive and specific detection of MPXV clade I, MPXV clade II, and GTPV for the first time. This assay serves as a prospective technological reserve to prevent MPXV from spilling over to sheep, thereby safeguarding both human and animal public health by mitigating the risk posed by sheep as a potential vector.

## Data Availability

The datasets presented in this study can be found in online repositories. The names of the repository/repositories and accession number(s) can be found in the article/Supplementary material.
