# Supplementary material for: Development of a multiplex real-time PCR for the simultaneous detection of monkeypox virus clades I, II, and goatpox virus
Source: Front Vet Sci. 2024 Nov 14;11:1483653. doi: 10.3389/fvets.2024.1483653 (PMC11603957; doi:10.3389/fvets.2024.1483653)
Supplement: Supplementary file 1 [file Image_1.pdf]

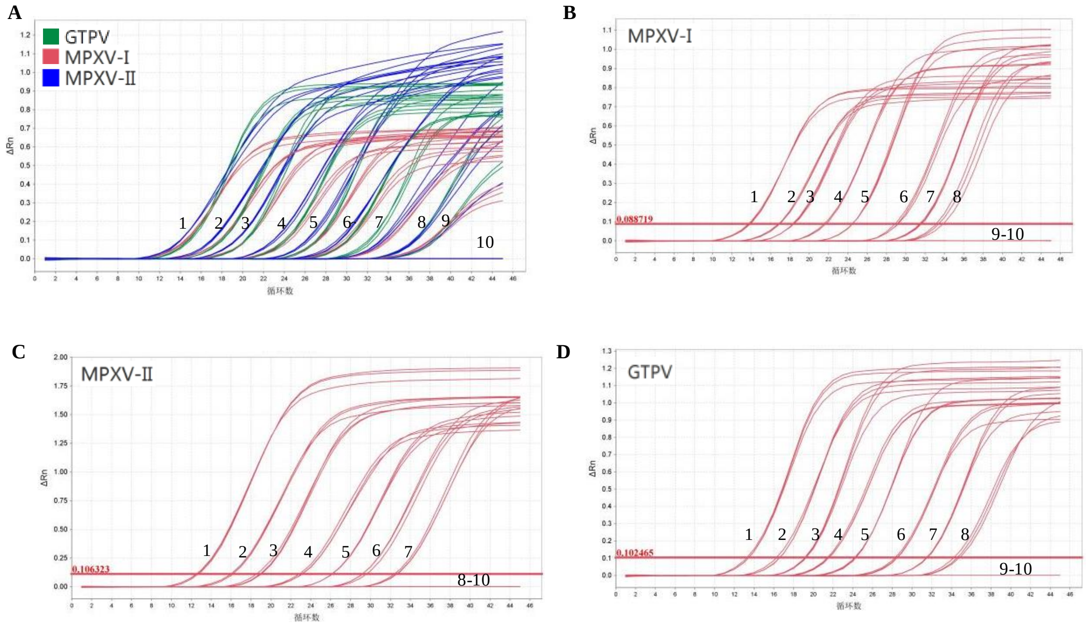

Figure S1 The sensitivity verification of the singleplex setting and the multiplex setting. Amplification curve **(A)** for GTPV, MPXV clade I and MPXV clade II, amplification curve **(B)** for MPXV clade I, amplification curve **(C)** for MPXV clade II and amplification curve **(D)** for GTPV. 1-9: Final concentration of standard plasmid 1.5×10^{7}~1.5×10^{-1}copies /μL, 10: negative control (no enzyme water).

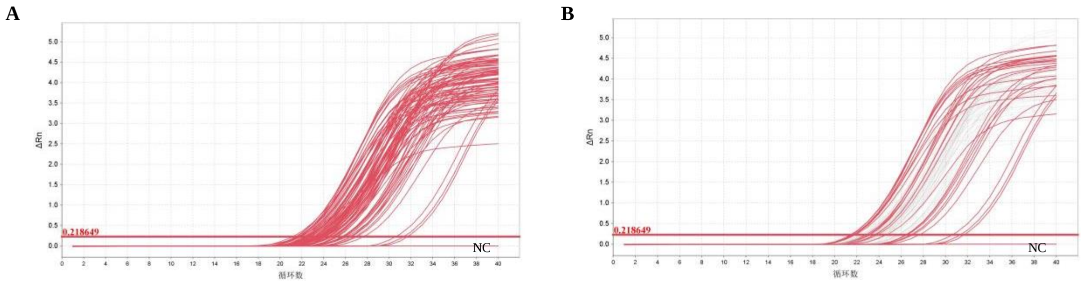

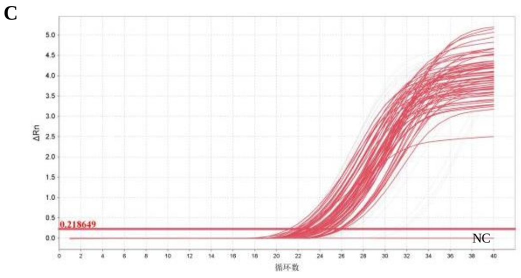

GAPDH-F	GCAAGTTCCACGGCACAG
GAPDH-R	GGTTCACGCCCATCACAA

Figure S2 The internal control detection result diagrams for each negative result **(A)**. Amplification curve **(B)** for the GAPDH gene in sheep tissues, amplification curve **(C)** for the GAPDH gene in sheep nasal swabs. Negative control(NC) (no enzyme water).
